# The mediating effect of serum cortisol between stigma and post-stroke depression in stroke patients

**DOI:** 10.3389/fpubh.2025.1682528

**Published:** 2025-10-06

**Authors:** Shitong Gong, Shiyan Wang, Jiangbo Wang, Yiming Yin, Yihao Wu

**Affiliations:** Xuzhou Central Hospital, XuZhou Clinical School of Xuzhou Medical University, Xuzhou, Jiangsu, China

**Keywords:** post-stroke depression, stigma, serum cortisol, mediating effect, stroke

## Abstract

**Objective:**

This study aimed to explore the mediating effect of serum cortisol on the relationship between stigma and post-stroke depression (PSD) in patients with acute ischemic stroke. To enhance early screening for post-stroke depression and prevent its development.

**Methods:**

A total of 367 patients admitted to the Department of Neurology and Neurosurgery at Xuzhou Central Hospital between January and December 2024 were selected using a convenience sampling method. Participants completed a general information questionnaire and the 8-item Stigma Scale for Chronic Illness, and their serum cortisol levels were measured at 8:00 a.m. the day after admission. Spearman correlation was used to analyze the correlation between serum cortisol, stigma level and depression degree in patients with acute ischemic stroke. The mediating effect Model was tested by Model 4 model in the PROCESS plug-in.

**Results:**

Among the participants, 182 were in the PSD group and 185 in the non-PSD group, with significant differences in income, education, serum cortisol, and stigma levels between the groups (*p* < 0.05). Spearman correlation analysis showed a significant positive correlation between stigma and depression severity (*r* = 0.715, *p* < 0.001), stigma and serum cortisol (*r* = 0.193, *p* < 0.001), and serum cortisol and depression severity (*r* = 0.261, *p* < 0.001). Mediation analysis using Model 4 of the PROCESS macro indicated that serum cortisol partially mediated the relationship between stigma and depression, with a mediating effect size of 0.019 (95%*CI*: 0.004–0.046), accounting for 2.5% of the total effect.

**Conclusion:**

These findings suggest that serum cortisol plays a partial mediating role between stigma and PSD in patients with acute ischemic stroke, highlighting a potential biological mechanism linking psychosocial stress to mental health outcomes in this population.

## Introduction

Stroke is a condition characterized by high morbidity, mortality, and disability rates. Post-stroke depression (PSD) is among the most common and severe neuropsychiatric complications of stroke. Although PSD was first recognized by psychiatrists over a century ago (1), systematic research on the condition did not begin until the 1970s. PSD typically presents with more severe depressive symptoms than general depression. Its core manifestations include a persistently low mood accompanied by cognitive impairment, apathy, anhedonia, loss of appetite, sleep disturbances, fatigue, self-blame, self-harming behavior, and even suicidal ideation.

Compared to stroke patients without depressive symptoms, those with PSD experience a significantly lower quality of life, higher mortality rates, and an increased risk of stroke recurrence. Despite the rising incidence of PSD in recent years, public awareness, particularly of its early stages, remains limited. Jorgensen et al. (2) analyzed data from 157,243 eligible stroke patients between January 2001 and December 2011 using Cox proportional hazards regression. They found that 25.4% of patients developed PSD within 2 years of stroke onset, yet only 7.8% recognized their depressive symptoms. These individuals often did not receive timely or standardized treatment, allowing psychological symptoms to progress despite improvements in physical function, thereby impeding their overall rehabilitation.

The onset of PSD is typically the result of a combination of neurobiological and psychosocial factors. One of the most well-established biological mechanisms is the hypothalamic–pituitary–adrenal (HPA) axis, a key neuroendocrine stress response system that regulates mood, immunity, and metabolism (3). HPA axis hyperactivity has been consistently linked to the pathogenesis of depression. Glucocorticoids (GCs) and their receptors are critical components of this axis, and numerous studies have identified a strong correlation between elevated cortisol levels and the development of depression (4–8).

In addition to biological mechanisms, psychosocial factors, particularly stigma, play an essential role in the emergence of PSD. Some scholars have proposed the psychosocial vulnerability model, which posits that depressive symptoms during stroke rehabilitation are initially triggered by a decline in activities of daily living. A subsequent lack of family and social support may then contribute to the development of acute post-stroke depression (PSD) (9). If functional disabilities persist, the ongoing absence of meaningful social connections can lead to chronic PSD. The concept of stigma was first introduced by sociologist Goffman in 1969, who defined it as “attributes that severely damage an individual’s social identity” (10). He categorized stigma into two main types: “internalized or perceived stigma” and “enacted stigma.” In 2012, Molina et al. (11) developed the 8-item version of the Stigma Scale for Chronic Illness (SSCI-8). Later, Deng Cuiyu et al. (12) translated and validated a Chinese version of the scale. In a 2017 study, Fred Stephen Sarfo et al. reported that up to 80% of stroke survivors in West Africa experienced varying degrees of stigma (13), which negatively affected both their physical recovery and mental well-being. As the intensity of perceived stigma increases, patients are more likely to suffer from anxiety, depression, and related emotional disturbances. In recent years, studies by Chinese scholars have further explored stigma in stroke patients, demonstrating a negative correlation between stigma severity and both overall hope levels and self-esteem scores. Reduced hope and diminished self-esteem may increase susceptibility to post-stroke depression (14).

This study aims to investigate the mediating role of serum cortisol in the relationship between stigma and PSD. By examining cortisol levels, perceived stigma, and depressive symptoms in stroke patients, this research seeks to identify more effective intervention points to enhance their quality of life.

## Materials and methods

### Subjects

A total of 426 patients with acute stroke admitted to the Department of Neurology and Neurosurgery of Xuzhou Central Hospital from January 2024 to December 2024 were initially selected; after excluding 48 patients who could not cooperate with psychological assessment and 11 patients who died or were lost to follow-up, 367 patients with acute stroke were finally included in the study.

Inclusion criteria: The inclusion criteria for this study were as follows: (1) patients met the diagnostic criteria outlined in the “Guidelines for the diagnosis and Treatment of acute ischemic Stroke in China”; (2) diagnosis was confirmed by brain Computed Tomography or Magnetic Resonance Imaging; (3) patients were able to cooperate in completing the scale examination; (4) signed informed consent was obtained; and (5) patients were prescribed at least one long-term medication (used for more than 3 months) at the time of discharge, indicative of the therapeutic burden associated with their clinical condition. Notably, none of the patients were undergoing treatment with antidepressant agents. The exclusion criteria included: (1) age below 18 years; (2) inability to undergo psychological assessment due to severe stroke, cognitive impairment, aphasia, or dysarthria; (3) a prior diagnosis of depression or presence of depressive symptoms before stroke; (4) history of Cushing’s syndrome, adrenal hyperplasia, or tumors; (5) history of hepatitis, tuberculosis, or other infectious diseases; (6) history of central nervous system (CNS) infections, dementia, schizophrenia, or other mental disorders; and (7) life expectancy of less than 3 months or inability to complete follow-up for other reasons. This study was approved by the Ethics Committee of Xuzhou Central Hospital (Approval No.: XZXY-LK-20240731-0112).

### Survey tools

#### General data collection

After admission, general patient data were collected, including age, gender, marital status, employment status, income level, education level, type of medical insurance, personal medical history, and past medical history.

#### Serum cortisol test

The fasting venous blood sample of 3 mL was collected at 8 a.m. on the day following admission, centrifuged at 3400 RPM for 5 min, and the serum cortisol level was measured using the IMMULITE 2000 automatic electrochemiluminescence analyzer along with its supporting reagents.

### Clinical evaluation

#### Stigma assessment

The assessment of stroke-related stigma was conducted by two neurologists in a quiet setting after patient admission, using the SSCI-8 as a self-evaluation tool. The SSCI-8, adapted by Deng Cuiyu et al. from the Chinese version of the SSCI-24 (12), consists of 8 items, 3 measuring internalized stigma and 5 measuring external stigma, with all items positively worded. Each item is rated on a 5-point Likert scale: “never,” “rarely,” “sometimes,” “often,” and “always,” scored from 1 to 5. The total score ranges from 5 to 40, with higher scores indicating higher levels of perceived stigma. The scale demonstrated strong internal consistency, with a Cronbach’s *α* coefficient of 0.892, and good test–retest reliability at 0.809, indicating that it is a reliable, valid, and stable instrument for evaluating stigma in stroke patients.

#### Assessment of depression

The Hamilton Depression Scale-24 (HAMD-24), developed by Hamilton in 1960, was used in this study to assess the severity of depressive symptoms. This scale is widely recognized for its simplicity and effectiveness in evaluating the degree of depression, with higher total scores indicating more severe depressive symptoms. The HAMD-24 version includes 24 items and can be categorized into seven symptom clusters: anxiety/somatization, weight, cognitive impairment, diurnal variation, sleep disorder, and hopelessness. In this study, patients were followed up at 3 months, two neurologists conducted the assessments independently in a quiet environment, and the final scores were calculated as the average of their evaluations.

### Statistical analysis

**Figure 1:**
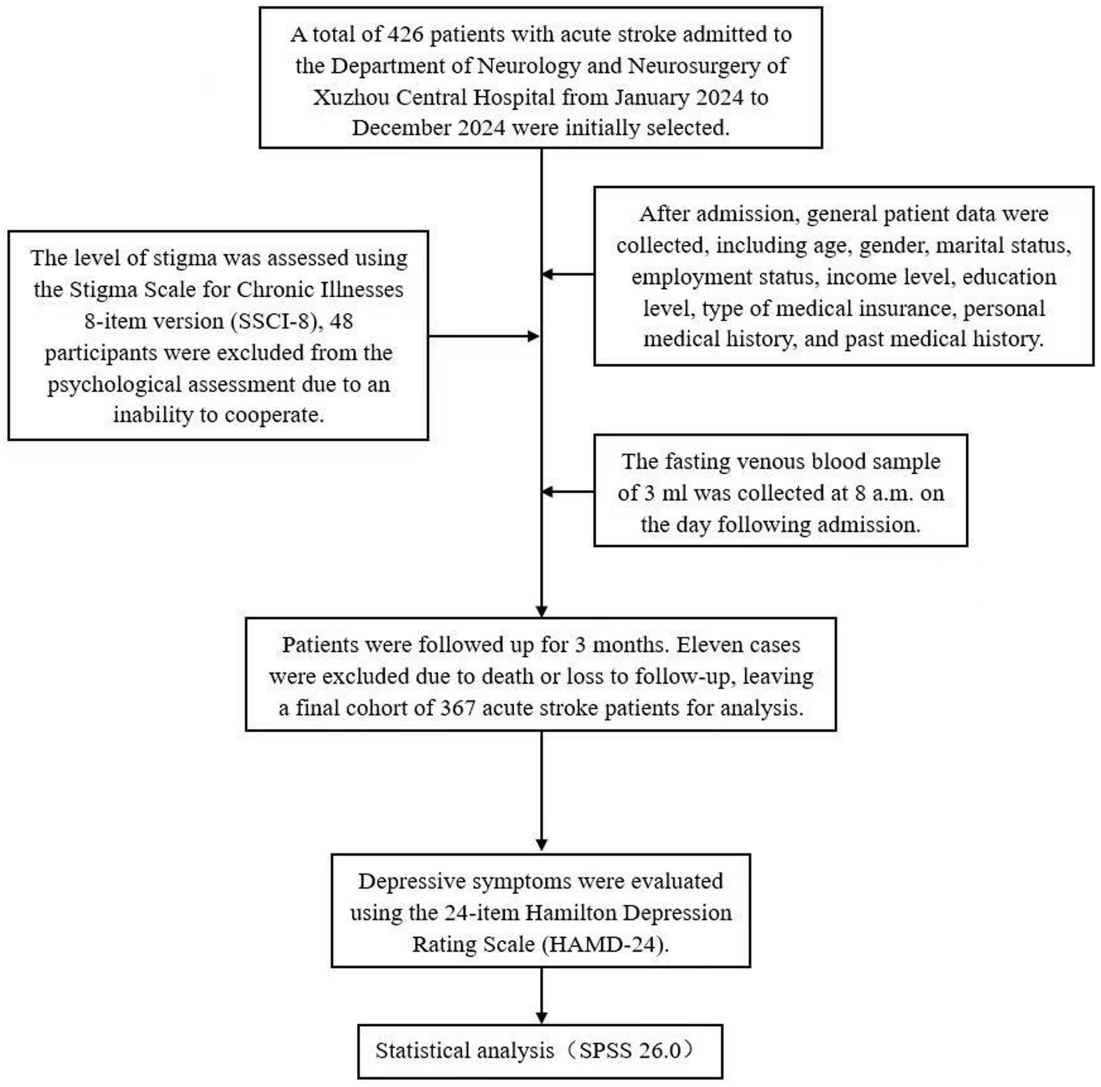
Study flowchart.

SPSS 26.0 software was used for data analysis. Measurement data were first tested for normality; those conforming to a normal distribution were presented as mean ± standard deviation (x̄ ± s), and comparisons between groups were conducted using independent sample t-tests. Data not meeting normal distribution criteria were expressed as median (interquartile range), and the Mann–Whitney U test was used for group comparisons. Categorical data were expressed as the number of cases (percentage), with unordered categorical data compared using the chi-square test and ordered categorical data compared using the Mann-Whitney U test. Spearman correlation analysis was used to assess correlations among indicators, while logistic regression analysis was conducted to identify factors associated with PSD. A *p*-value of less than 0.05 was considered statistically significant (Figure 1).

## Results

### General data analysis

The demographic data analysis results of the two groups are presented in Table 1. Independent sample t-test results indicated no statistically significant differences in age and BMI between the PSD and non-PSD groups (*p* > 0.05). Chi-square test results showed no significant differences in gender, marital status, education level, income, employment status, or type of medical insurance between the two groups (*p* > 0.05). However, the proportion of patients with a monthly income below 5,000 yuan and those with an education level of senior high school or above was significantly higher in the PSD group compared to the non-PSD group, and these differences were statistically significant (*p* ≤ 0.05).

**Table 1:** Analysis of demographic data of the two groups.

Baseline data	Overall (*n* = 367)	Non-PSD group (*n* = 185)	PSD group (*n* = 182)	*T/χ*	*p*
Age (years)	63.20 ± 13.21	63.12 ± 12.95	63.29 ± 13.51	0.121	0.904
Gender [example (%)]				0.051	0.821
Male	234 (63.8)	119 (64.3)	115 (63.2)		
Female	133 (36.2)	66 (35.7)	67 (36.8)		
BMI	25.23 ± 3.55	25.41 ± 3.15	25.04 ± 3.92	0.984	0.326
Marital status [example (%)]				0.120	0.729
Married	357 (97.3)	181 (97.8)	176 (96.7)		
Unmarried/divorced/widowed	10 (2.7)	4 (2.2)	6 (3.3)		
Work situation [example (%)]				1.523	0.467
Job	169 (46.0)	89 (48.1)	80 (44.0)		
Retire/take a leave	114 (31.1)	52 (28.1)	62 (34.1)		
No job	84 (22.9)	44 (23.8)	40 (22.0)		
Income situation [example (%)]				5.049	0.025^*^
<5,000	186 (50.7)	83 (44.9)	103 (56.6)		
≥5,000	181 (49.3)	102 (55.1)	79 (43.4)		
Level of education [example (%)]				11.510	0.001^**^
Junior high school and below	184 (50.1)	109 (58.9)	75 (41.2)		
High school and above	183 (49.9)	76 (41.1)	107 (58.8)		
Type of health insurance [example (%)]				0.658	0.714
Employees	272 (74.1)	135 (73.0)	137 (75.3)		
Residents	90 (24.5)	48 (25.9)	42 (23.1)		
Out of pocket	5 (1.4)	2 (1.1)	3 (1.6)		

^*^p ≤ 0.05, ^**^p ≤ 0.01.

### Analysis of stigma level of patients in two groups

The comparison results of the SSCI-8 scale between the two groups are shown in Table 2. The Mann-Whitney U test revealed that the total SSCI-8 score, as well as the intrinsic and extrinsic stigma scores, were significantly higher in the PSD group compared to the non-PSD group, with all differences reaching statistical significance (*p* ≤ 0.05).

**Table 2:** Comparison of SSCI-8 scale between the two groups [M (Q25, Q75)].

Groups	Number of cases	SSCI-8 total score (points)	Intrinsic stigma (points)	Extrinsic stigma (points)
Overall	367	16.00 (13.00, 20.00)	9.00 (6.00, 12.00)	8.00 (6.00, 9.00)
Non-PSD group	185	13.00 (11.00, 16.00)	6.00 (5.00, 7.00)	6.00 (5.00, 8.50)
PSD group	182	20.00 (17.00, 24.00)	11.00 (10.00, 14.00)	8.00 (6.00, 10.25)
*Z*	–	−13.251	−14.252	−7.184
*p*	–	<0.001^**^	<0.001^**^	<0.001^**^

^*^p ≤ 0.05, ^**^p ≤ 0.01.

The results of the stratified comparison of stigma levels between the two groups of patients are shown in Table 3. The *χ2* test results indicated that the proportion of patients with moderate to severe stigma was significantly higher in the PSD group compared to the non-PSD group. Conversely, the proportion of patients with no stigma or mild stigma was significantly lower in the PSD group, with statistically significant differences (*p* ≤ 0.05). Pairwise comparisons indicated significant differences between no stigma and mild stigma, moderate stigma and severe stigma (*p* ≤ 0.05). Additionally, significant differences were observed between mild and moderate/severe stigma (*p* ≤ 0.05). However, no significant difference was found in the risk of PSD between patients with moderate and severe stigma.

**Table 3:** Comparison of stigma levels between two groups.

Stigma levels [*n* (%)]	No_a_	Mild_b_	Moderate_c_	Severe_c_
Overall (*n* = 367)	73 (19.9)	197 (53.7)	86 (23.4)	11 (3.0)
Non-psd group (*n* = 185)	69 (37.3)	111 (60.0)	5 (2.7)	0 (0)
PSD group (*n* = 182)	4 (2.2)	86 (47.3)	81 (44.5)	11 (6.0)
*χ^2^*	139.197			
*p*	<0.001^**^			

^*^p ≤ 0.05, ^**^p ≤ 0.01.

a,b,c: If the marked letters between the two groups are the same, it indicates that there is no statistically significant difference between the two groups. If the marked letters between the two groups are different, it indicates that the difference between the two groups is statistically significant.

### Analysis of serum cortisol level of patients in two groups

The comparison results of serum cortisol levels between the two groups are shown in Table 4, indicating that the serum cortisol level in the PSD group was significantly higher than that in the non-PSD group, with the difference being statistically significant (*p* ≤ 0.05).

**Table 4:** Comparison of serum cortisol levels between the two groups [M (Q25, Q75)].

	Overall (*n* = 367)	Non-psd group (*n* = 185)	PSD group (*n* = 182)	*Z*	*p*
Serum cortisol (μg/dL)	11.30 (8.25, 15.48)	10.20 (7.38, 12.97)	13.10 (9.32, 16.81)	−5.010	<0.001^**^

^*^p ≤ 0.05, ^**^p ≤ 0.01.

### Correlation analysis of stigma level with depression and serum cortisol

**Figure 2:**
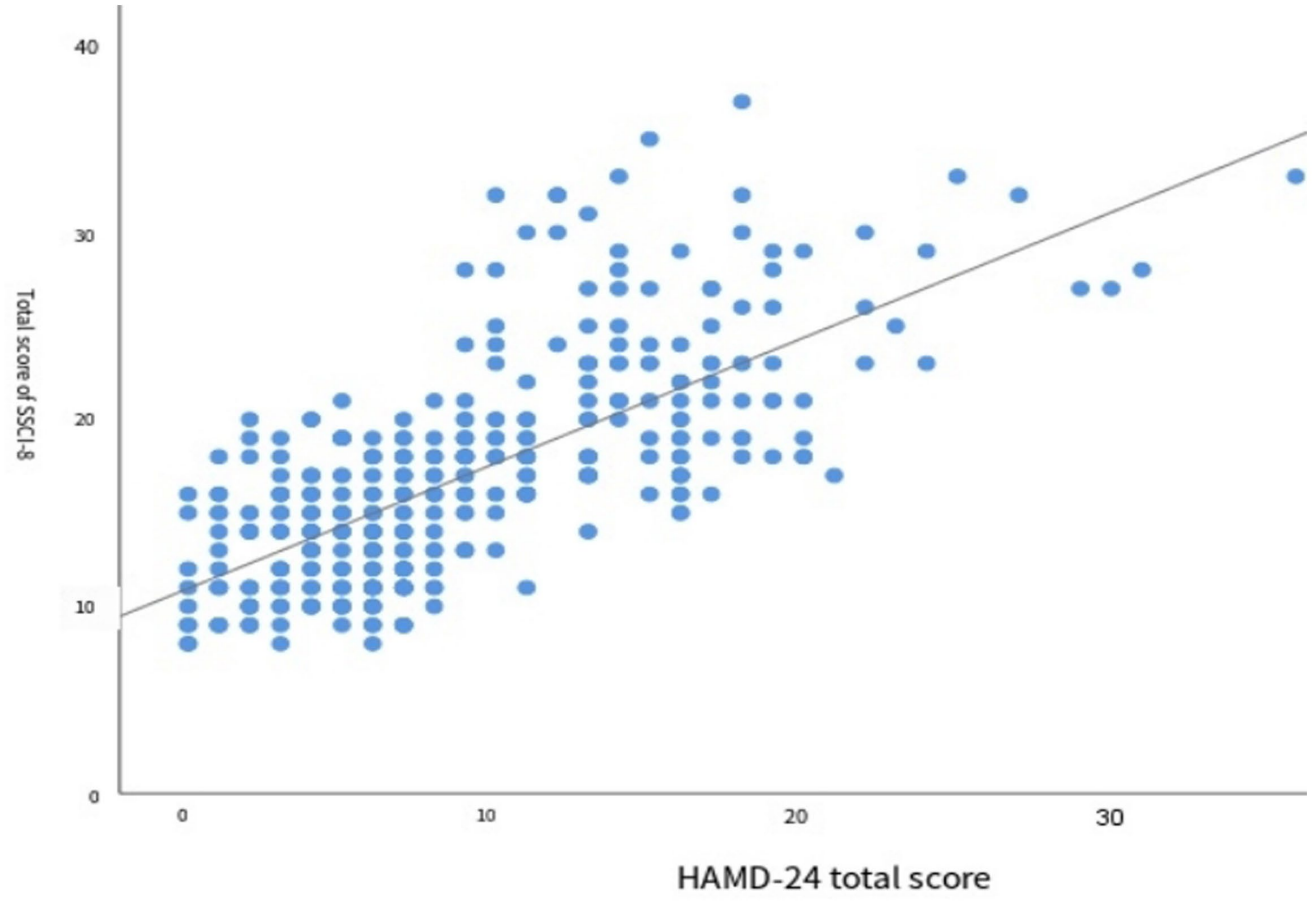
Scatter plot of the total score of SSCI-8 and HAMD-24.

The results of correlation analysis between the total score of the SSCI-8 scale and the degree of depression are shown in Figure 2. Spearman correlation analysis revealed a significant positive correlation between the two variables (*r* = 0.715,*p* < 0.001), indicating that higher levels of stigma were associated with greater severity of depression.

**Figure 3:**
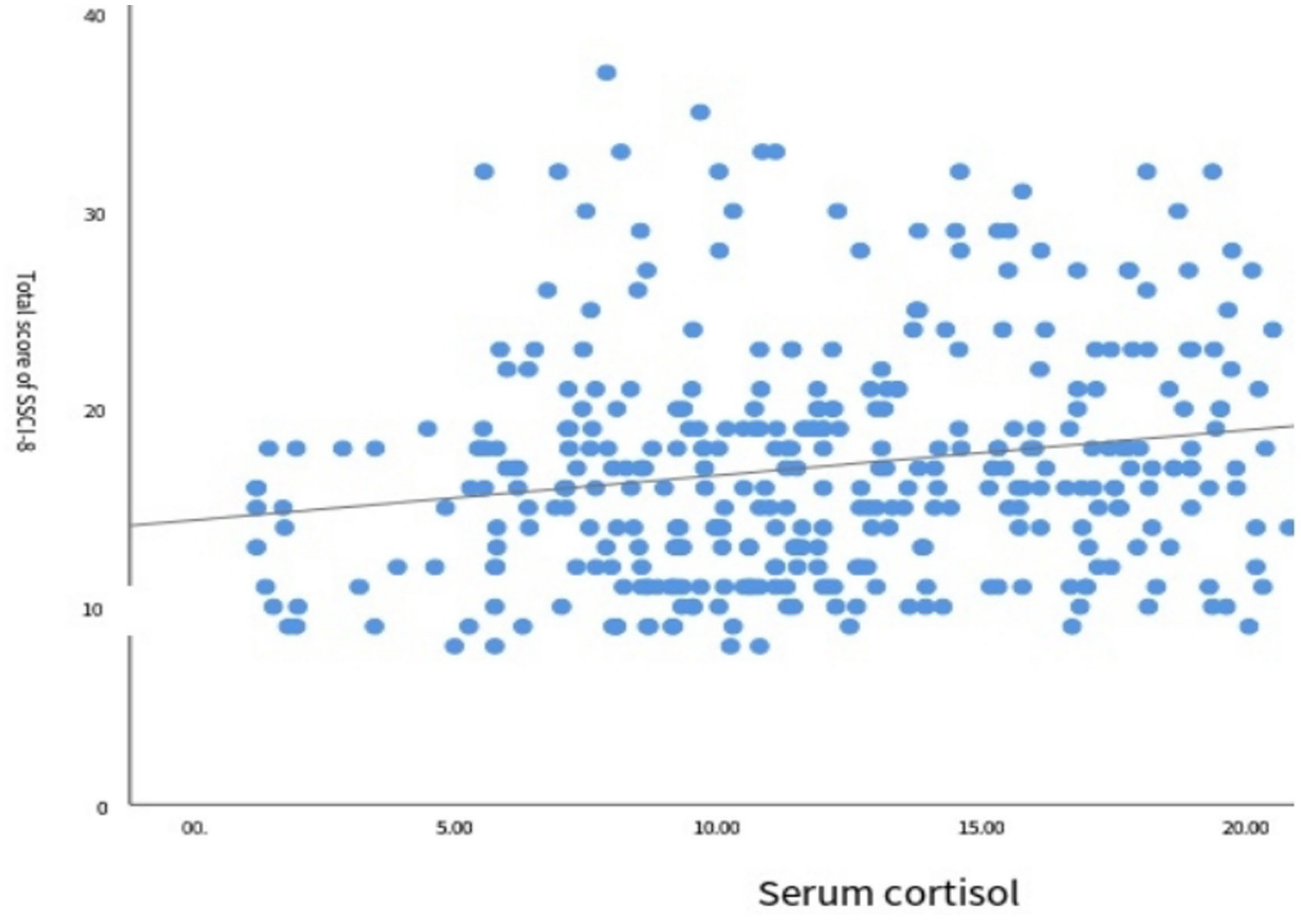
Scatter plot of SSCI-8 total score and serum cortisol.

The results of correlation analysis between the SSCI-8 scale score and serum cortisol levels are shown in Figure 3. Spearman correlation analysis indicated a positive correlation between the two variables (*r* = 0.193, *p* < 0.001), suggesting that higher stigma scores were associated with increased serum cortisol levels.

**Figure 4:**
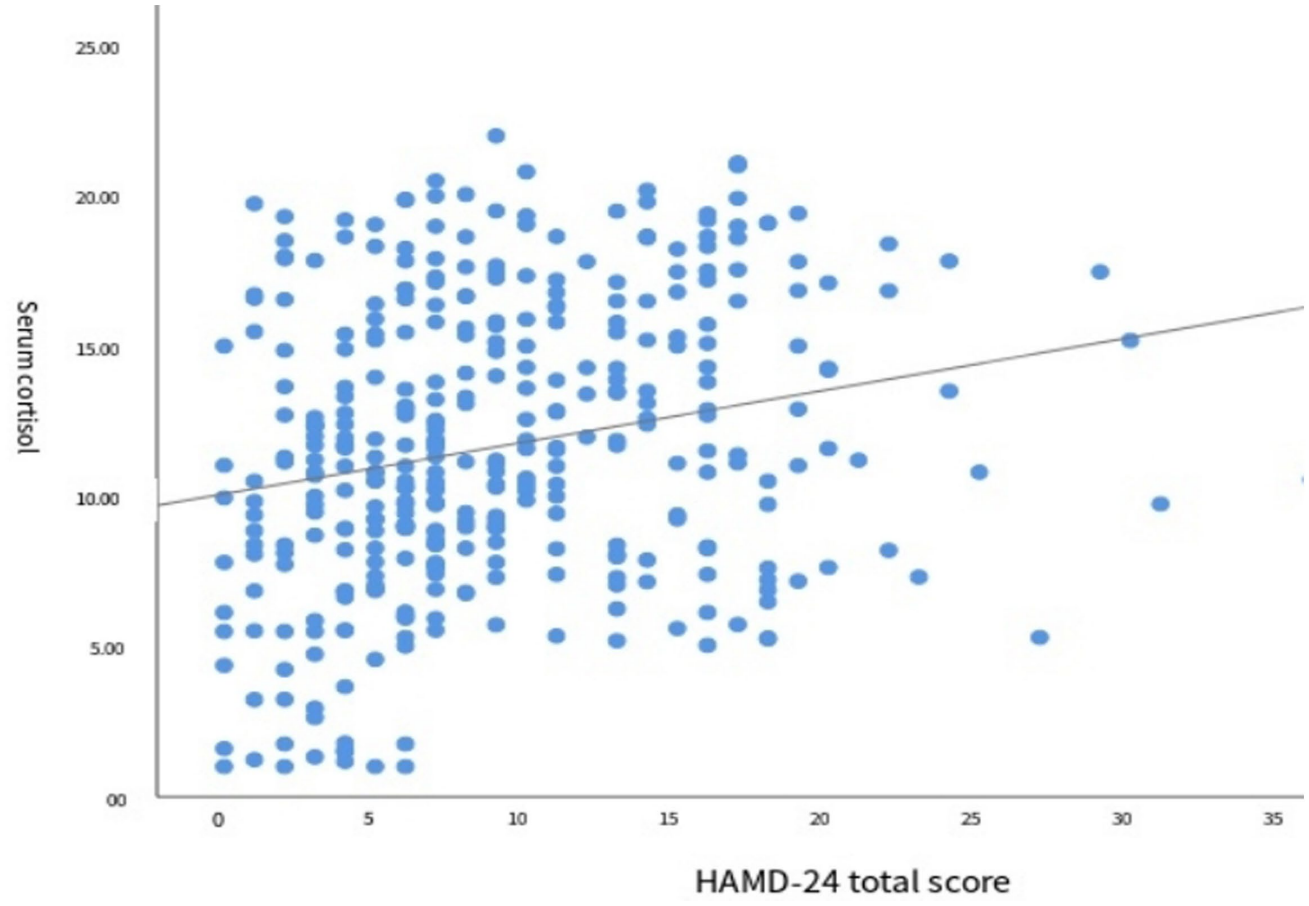
Scatter plot of serum cortisol and HAMD-24 total score.

The results of the correlation analysis between serum cortisol levels and the degree of depression are shown in Figure 4. Spearman correlation analysis revealed a positive correlation between the two variables (*r* = 0.261, *p* < 0.001), indicating that higher serum cortisol levels were associated with greater severity of depression (Table 5).

**Table 5:** Correlation of stigma level, serum cortisol level and depression level in stroke patients (*r* value).

Items	Level of stigma	Serum cortisol	Depression levels
Level of stigma	1		
Serum cortisol	0.193^**^	1	
Level of depression	0.715^**^	0.261^**^	1

^*^p ≤ 0.05, ^**^p ≤ 0.01.

### The mediating relationship between serum cortisol and stigma and PSD

**Figure 5:**
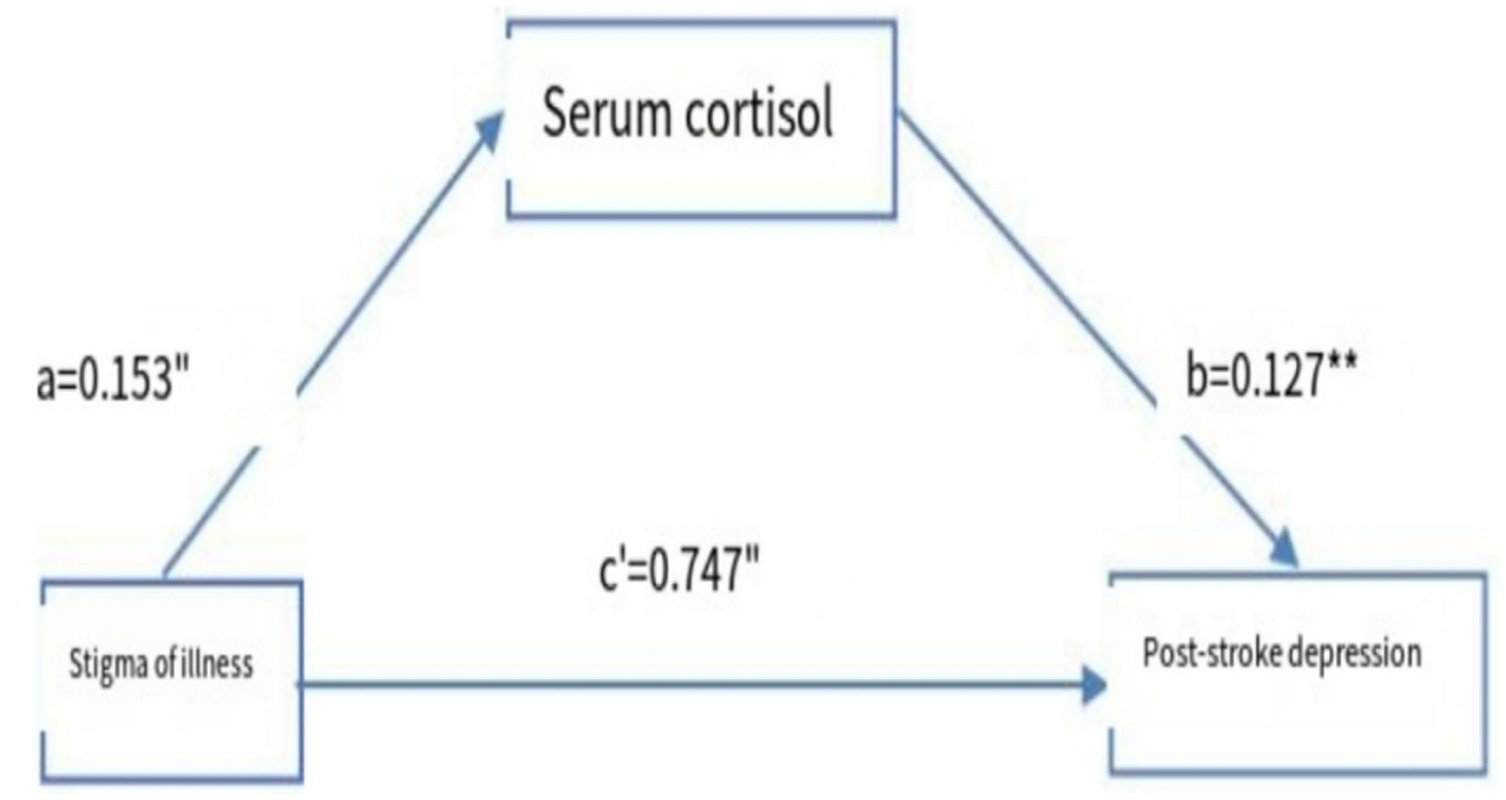
Model of the mediating effect of serum cortisol in stroke patients between stigma and PSD. **a** and **b** are the mediating variables of the self-efficacy; **c** represents the direct; “*p*<0.05”, “*p*<0.01”.

Model 4 (simple mediation model) of the PROCESS plug-in in SPSS software was used to examine whether serum cortisol mediates the relationship between stigma level and PSD. As shown in Table 6, stigma significantly predicted PSD (path c) with *Z* = 19.655 and *p* < 0.001. After including serum cortisol in the model, the direct effect of stigma on PSD (path c′) remained significant (*Z* = 18.973, *p* < 0.001). Furthermore, the 95% confidence intervals for both the direct effect of stigma on PSD and the mediating effect of serum cortisol did not include zero (Table 7), indicating statistical significance. These results demonstrate that stigma not only directly predicts PSD but also indirectly affects it through the mediating role of serum cortisol. The direct and mediating effects were 0.750 and 0.017, respectively. The relationships among the variables are illustrated in Figure 5.

**Table 6:** Results of mediating model test of serum cortisol between stigma and PSD in stroke patients.

Outcome variables	Predictor variables	Fit metrics		Coefficient significance	
		*R^2^*	*F*	*t/Z*	*p*
PSD	Stigma (c)	0.514	386.334	19.655	<0.001^**^
Serum cortisol	Stigma (a)	0.035	13.237	3.638	<0.001^**^
PSD		0.523	199.707		
	Serum cortisol (b)			2.621	0.009^**^
	Stigma (c ‘)			18.973	<0.001^**^

^*^p ≤ 0.05, ^**^p ≤ 0.01.

**Table 7:** Breakdown table of total effect, direct effect and mediating effect of stigma on PSD.

Items	Effect size	Standard error of indirect effect	95% confidence interval		Relative effect size
			Lower limit	Upper limit	
Total effect	0.766	0.039	0.690	0.843	
Direct effect	0.747	0.039	0.670	0.825	97.5%
Indirect effects	0.019	0.010	0.004	0.046	2.5%

## Discussion

### Relationship between PSD and cortisol

Previous studies have shown that patients with depression tend to have higher salivary (15) or hair (16) cortisol levels than non-depressed individuals; however, there is limited research on the relationship between serum cortisol levels and PSD. Although serum cortisol testing is invasive, its concentration is typically higher than that found in saliva or hair, making changes easier to detect at an earlier stage, an advantage for the timely identification and diagnosis of PSD. In this study, serum cortisol was used as an observational marker. The results showed that cortisol levels in the PSD group were significantly higher than in the non-PSD group. This may be attributed to the role of the HPA axis, the body’s primary neuroendocrine stress response system, which regulates mood, immunity, and metabolism. Upon receiving signals from the hippocampus or other regions, the hypothalamus releases corticotropin-releasing hormone (CRH) from its paraventricular nucleus, which stimulates the pituitary gland to secrete adrenocorticotropic hormone (ACTH). ACTH then promotes the synthesis and release of GCs from the adrenal cortex (17). GCs and their receptors are vital components of the HPA axis.

After a stroke, the HPA axis can become overactivated. Due to impaired negative feedback mechanisms, the levels of CRH, ACTH, and cortisol may fail to reach equilibrium, resulting in excessive cortisol production and accumulation (18). Elevated cortisol levels can adversely affect the CNS. One study observed that patients with long-term exposure to exogenous cortisol exhibited neurobiological changes (18) such as hippocampal volume reduction, prefrontal cortex atrophy, and dendritic hypertrophy in the amygdala. These changes contribute to reduced neurogenesis and neuronal survival, dysregulation of the neurotrophic system, and a decline in 5-hydroxytryptamine neurotransmitter levels, thereby increasing the risk of PSD. A 2017 study by Ferrari et al. (19) further confirmed that HPA axis dysfunction is associated with an increased risk of PSD. The HPA axis also interacts with the serotonergic system (20) by binding to the serotonin-transporter-linked polymorphic region (5-HTTLPR). Prolonged exposure to high cortisol levels can alter the polymorphism of 5-HTTLPR, modifying the biological stress response and further exacerbating the severity of PSD. Therefore, early detection of serum cortisol levels after stroke may be a valuable tool for identifying patients at high risk of developing PSD and can facilitate timely intervention.

### The correlation between PSD and stigma level

In this study, the total stigma score, as well as the scores for internalized and enacted stigma, were significantly higher in the PSD group compared to the non-PSD group. A correlation analysis between the SSCI-8 (stigma) score and the HAMD-24 (depression) score revealed a strong positive correlation (*r* = 0.715, *p* < 0.01), indicating that higher levels of stigma were associated with greater severity of depression in patients with PSD. This finding raises the question of whether varying levels of stigma are associated with differing risks of PSD. To explore this, the level of stigma was stratified based on SSCI-8 scores: 8–11 points were categorized as “no stigma,” 12–20 as “mild stigma,” 21–31 as “moderate stigma,” and 32–40 as “severe stigma.” The results showed that the overall stigma level among stroke patients was predominantly mild to moderate. Patients in the non-PSD group were mostly in the “no stigma” or “mild stigma” categories, while those in the PSD group primarily exhibited mild to moderate stigma. Importantly, the level of stigma in the PSD group was significantly higher than that in the non-PSD group. Furthermore, patients with moderate or severe stigma had a significantly higher risk of developing PSD compared to those with mild or no stigma.

Stigma significantly impacts social participation, with patients experiencing higher levels of stigma exhibiting lower degrees of engagement in social activities. The perception of stigma and experiences of discrimination can lead stroke survivors to withdraw from social interactions, resulting in increased social isolation and reluctance to reintegrate into society (21). Moreover, among patients with physical disabilities living in the community, stigma contributes to a diminished self-concept. In this study, all 11 stroke patients identified with severe stigma were also diagnosed with PSD. During evaluation and follow-up, these patients consistently exhibited strong self-denial. Within the context of traditional Chinese cultural values, such self-denial may lead patients to view themselves as burdens who are unable to support their families, further intensifying feelings of worthlessness. Additionally, these individuals often demonstrate poor treatment adherence. According to the psychological cognitive model developed by Corrigan et al. (22), stigma can negatively influence patients’ cognition, behavior, and emotional responses, leading some to refuse medication or rehabilitation interventions. This is particularly concerning for patients with persistent sequelae, as prolonged rehabilitation and unsatisfactory outcomes further elevate the risk of PSD. Lu et al. (23) conducted a study on Chinese stroke survivors in the community and found that the level of stigma varied according to the type of post-stroke sequelae. Patients with hemiplegia, dysphagia, facial paralysis, and aphasia reported higher levels of stigma, and the degree of stigma increased with the number of sequelae. Therefore, for stroke patients with severe neurological deficits and reduced quality of life, special attention should be given to their perceived stigma and its association with PSD risk and depression severity. Patients exhibiting moderate to severe levels of stigma require closer psychological monitoring, as they are at significantly higher risk of developing PSD.

### The mediating effect of serum cortisol on stigma and PSD

This study demonstrates that stigma not only directly predicts the occurrence of PSD but also exerts an indirect effect through the mediating role of serum cortisol, suggesting that serum cortisol levels partially mediate the relationship between stigma and PSD. This finding provides important clinical evidence supporting the combined use of stigma severity and serum cortisol levels for early identification and prediction of PSD in stroke patients. Individuals experiencing stigma often have a diminished self-concept and perceive themselves as burdens to their families, leading to chronic psychological stress. Previous research has shown that the HPA axis typically responds to stress through a negative feedback mechanism (23, 24). While circadian fluctuations and stress responses of the HPA axis are usually adaptive, long-term exposure to stigma may disrupt this regulation, resulting in a flattened diurnal cortisol slope or abnormal cortisol arousal responses. Such dysregulation increases vulnerability to mood disorders, including depression and anxiety. Additionally, external stigma, reflected through perceived discrimination or social rejection, further intensifies stress. A meta-analysis by Busse et al. (25) involving 27 studies confirmed that discriminatory experiences are linked to HPA axis dysfunction, and the duration of such experiences may explain their long-term physiological and psychological consequences. Acute stroke is a sudden negative event, and its long-term sequelae significantly impair quality of life and social functioning, increasing the likelihood of stigma, prolonged stress, HPA axis dysregulation, and ultimately PSD. Therefore, assessing both stigma and serum cortisol levels in stroke patients can aid in the timely identification of individuals at high risk of developing PSD and facilitate early intervention strategies to potentially reduce its incidence.

### Limitations of this study

There are some limitations to this study. First, as a single-center investigation, it involved a relatively limited patient population, which may restrict the generalizability of the findings. The potential influences of dietary habits, regional variations, lifestyle differences, and socioeconomic factors therefore remain unclear. Second, the assessment of depressive symptoms was conducted only at a single time point (3 months post-stroke), thereby lacking longitudinal observation of dynamic changes in depression levels over time. Third, cortisol measurement was performed only at 8 a.m., without assessing diurnal rhythm variations, which limits the interpretation of hypothalamic–pituitary–adrenal axis activity.

### Future research direction

Future studies should incorporate comparisons of different interventions for PSD and stigma to identify optimal clinical strategies and further elucidate the effect of stigma reduction on PSD.

## Summary

The experience of stigma can further diminish patients’ self-evaluation and social communication abilities, leading to self-doubt, social isolation, and ultimately the development of PSD. This highlights the significant predictive role of stigma in the onset of PSD. Additionally, cortisol may serve as a mediating factor, indicating that stigma may indirectly contribute to PSD through physiological pathways. The level of stigma among stroke patients is strongly associated with both the occurrence of PSD and the severity of depressive symptoms. Therefore, early clinical attention to stigma is essential. Rehabilitation programs for stroke patients should incorporate targeted interventions to reduce stigma, with the aim of restoring patients’ self-concept, enhancing psychological recovery, and ultimately decreasing the risk of PSD.

## Data Availability

The raw data supporting the conclusions of this article will be made available by the authors, without undue reservation.
